# Hyperangulated blades or direct epiglottis lifting to optimize glottis visualization in difficult Macintosh videolaryngoscopy: a non-inferiority analysis of a prospective observational study

**DOI:** 10.3389/fmed.2023.1292056

**Published:** 2023-11-30

**Authors:** Viktor A. Wünsch, Vera Köhl, Philipp Breitfeld, Marcus Bauer, Phillip B. Sasu, Hannah K. Siebert, Andre Dankert, Maria Stark, Christian Zöllner, Martin Petzoldt

**Affiliations:** ^1^Department of Anesthesiology, Center of Anesthesiology and Intensive Care Medicine, University Medical Center Hamburg-Eppendorf, Hamburg, Germany; ^2^Institute of Medical Biometry and Epidemiology, University Medical Center Hamburg-Eppendorf, Hamburg, Germany

**Keywords:** airway management, intubation, intratracheal, laryngoscopy, laryngoscopes, epiglottis

## Abstract

**Purpose:**

It is unknown if direct epiglottis lifting or conversion to hyperangulated videolaryngoscopes, or even direct epiglottis lifting with hyperangulated videolaryngoscopes, may optimize glottis visualization in situations where Macintosh videolaryngoscopy turns out to be more difficult than expected. This study aims to determine if the percentage of glottic opening (POGO) improvement achieved by direct epiglottis lifting is non-inferior to the one accomplished by a conversion to hyperangulated videolaryngoscopy in these situations.

**Methods:**

One or more optimization techniques were applied in 129 difficult Macintosh videolaryngoscopy cases in this secondary analysis of a prospective observational study. Stored videos were reviewed by at least three independent observers who assessed the POGO and six glottis view grades. A linear mixed regression and a linear regression model were fitted. Estimated marginal means were used to analyze differences between optimization maneuvers.

**Results:**

In this study, 163 optimization maneuvers (77 direct epiglottis lifting, 57 hyperangulated videolaryngoscopy and 29 direct epiglottis lifting with a hyperangulated videolaryngoscope) were applied exclusively or sequentially. Vocal cords were not visible in 91.5% of the cases with Macintosh videolaryngoscopy, 24.7% with direct epiglottis lifting, 36.8% with hyperangulated videolaryngoscopy and 0% with direct lifting with a hyperangulated videolaryngoscope. Conversion to direct epiglottis lifting improved POGO (mean + 49.7%; 95% confidence interval [CI] 41.4 to 58.0; *p* < 0.001) and glottis view (mean + 2.2 grades; 95% CI 1.9 to 2.5; *p* < 0.001). Conversion to hyperangulated videolaryngoscopy improved POGO (mean + 43.7%; 95% CI 34.1 to 53.3; *p* < 0.001) and glottis view (mean + 1.9 grades; 95% CI 1.6 to 2.2; *p* < 0.001). The difference in POGO improvement between conversion to direct epiglottis lifting and conversion to hyperangulated videolaryngoscopy is: mean 6.0%; 95% CI −6.5–18.5%; hence non-inferiority was confirmed.

**Conclusion:**

When Macintosh videolaryngoscopy turned out to be difficult, glottis exposure with direct epiglottis lifting was non-inferior to the one gathered by conversion to hyperangulated videolaryngoscopy. A combination of both maneuvers yields the best result.

**Clinical trial registration:**

ClinicalTrials.gov, NCT03950934.

## 1 Introduction

Videolaryngoscopy has gained worldwide acceptance for the management of difficult intubation (1–6) and there is growing evidence that it prevents failed intubation, hypoxemic events and accidental esophageal intubation while improving the glottis view (3). Routine use of videolaryngoscopy has been recommended whenever feasible (7). In the last few years, videolaryngoscopy became more universally available in many hospitals (8, 9) and a reliable universal classification for videolaryngoscopy -the VIDIAC score- has recently been introduced (10, 11).

The percentage of glottic opening (POGO) has been used since 1998 (12) to evaluate glottis exposure during laryngoscopy; it estimates the visible proportion of the distance between the interarytenoid notch and the anterior commissure and has been used as an outcome variable in numerous studies, meta-analysis (4, 13) and Cochrane reviews (3).

Many manufacturers provide videolaryngoscopes with either Macintosh-type blades, which still allow a direct view on the glottis, or hyperangulated blades or both. Hyperangulated blades, however, allow a better view beneath the epiglottis, but this might not necessarily translate into easier intubation (3, 13–15). With both methods, the epiglottis is typically lifted by point pressure on the hyoepiglottic ligament transmitted by the blade tip placed in the epiglottic vallecula (indirect epiglottis lifting) (11, 16, 17). This basic mechanism has already been described by Macintosh in 1943 (16).

Although not well reported in the scientific literature, direct epiglottis lifting (by placing the tip of the laryngoscope beneath the epiglottis) with a conventional or videolaryngoscope is a widely accepted alternative, especially in difficult cases and in pediatrics. This, in turn, might be inspired by the straight blade technique that relies on direct epiglottis lifting (18–23). However, direct lifting with Macintosh-type blades has not yet been recommended in guidelines and current data are very limited (17, 19). It is unknown if the epiglottis might be relevantly mechanically altered by direct lifting with a Macintosh blade. On the other hand, there is growing evidence that multiple laryngoscopy attempts are associated with an increased risk of adverse outcomes such as hypoxia, esophageal intubation or pulmonary aspiration (24).

Macintosh videolaryngoscopy is widely used in daily clinical practice in many institutions. But what are the options if Macintosh videolaryngoscopy turns out to be difficult as the glottis view is severely restricted? A current metanalysis did not reveal significant differences between hyperangulated and Macintosh videolaryngoscopes with regard to POGO (13, 25) and currently data underscoring a beneficial effect of direct epiglottis lifting with Macintosh videolaryngoscopes are very limited (17, 19). Epiglottis lifting techniques have been identified as the missing piece of the puzzle in assessing difficult airway management (11, 26). Although important for decision-making in time-critical situations, it is unknown if direct epiglottis lifting or conversion to hyperangulated videolaryngoscopy improves glottis visualization equally well.

The primary aim of this analysis was to determine whether improvement in POGO (12) achieved by direct epiglottis lifting is non-inferior to the one accomplished by a conversion to hyperangulated videolaryngoscopes in situations where Macintosh videolaryngoscopy proved to be difficult.

## 2 Methods

The Videolaryngoscopic Intubation and Difficult Airway Classification (VIDIAC) trial is a single-center prospective observational study performed in accordance with the Declaration of Helsinki. It was approved by the Ethics Committee of the Medical Association of Hamburg (PV5856, August 10, 2018, amendment August 12, 2019; chair: Prof. Dr. R. Stahl), and registered with ClinicalTrials.gov (identifier: NCT03950934). The present findings result from an non-inferior analysis of an independent dataset prospectively acquired within the VIDIAC study (11). Participants gave written informed consent. The design and reporting is adapted to the STROBE statement (27).

### 2.1 Patient allocation and data collection

Adults who presented at our Anesthesia Preassessment Clinic before elective ear, nose and throat or oral and maxillofacial surgery between April 1, 2019 and April 3, 2020 were assessed for eligibility. All patients received a structured preoperative airway risk assessment in accordance with standards laid out by the Department of Anesthesiology that comprises clinical history and physical examinations [such as the upper lip bite test, the simplified airway risk index and flexible nasendoscopy (28, 29) if appropriate] (30, 31). Details are reported elsewhere (11). Patients were checked for indicators of awake tracheal intubation considering predictors such as difficult tracheal intubation, suspected difficult facemask and/or supraglottic-airway ventilation, apnea intolerance and risk for aspiration (6).

Videolaryngoscopes with Macintosh-type blades (C-MAC™, Karl Storz, Tuttlingen, Germany) where used first-line in all participants. Indirect epiglottis lifting facilitated by point pressure on the hyoepiglottic ligament with the blade tip placed in the epiglottic vallecula was attempted first-line in all patients (16). There was no anesthesia management protocol. Anesthesia induction, patient positioning, tracheal intubation, the use of airway adjuncts, airway optimization maneuvers and conversion to different intubation techniques and devices, for example, direct epiglottis lifting hyperangulated blades (C-MAC™ D-BLADE, Karl Storz, Tuttlingen, Germany) or flexible bronchoscopes were left at the discretion of the anesthetist. According to our institutional standards, neuromuscular blocking agents were used in all patients. A large variety of different airway operators, trainees as well as very experienced consultants with different levels of professional experiences in anesthesiology were involved in this study in order to reflect representative real-world conditions (10, 11).

### 2.2 Eligibility criteria

**Figure 1:**
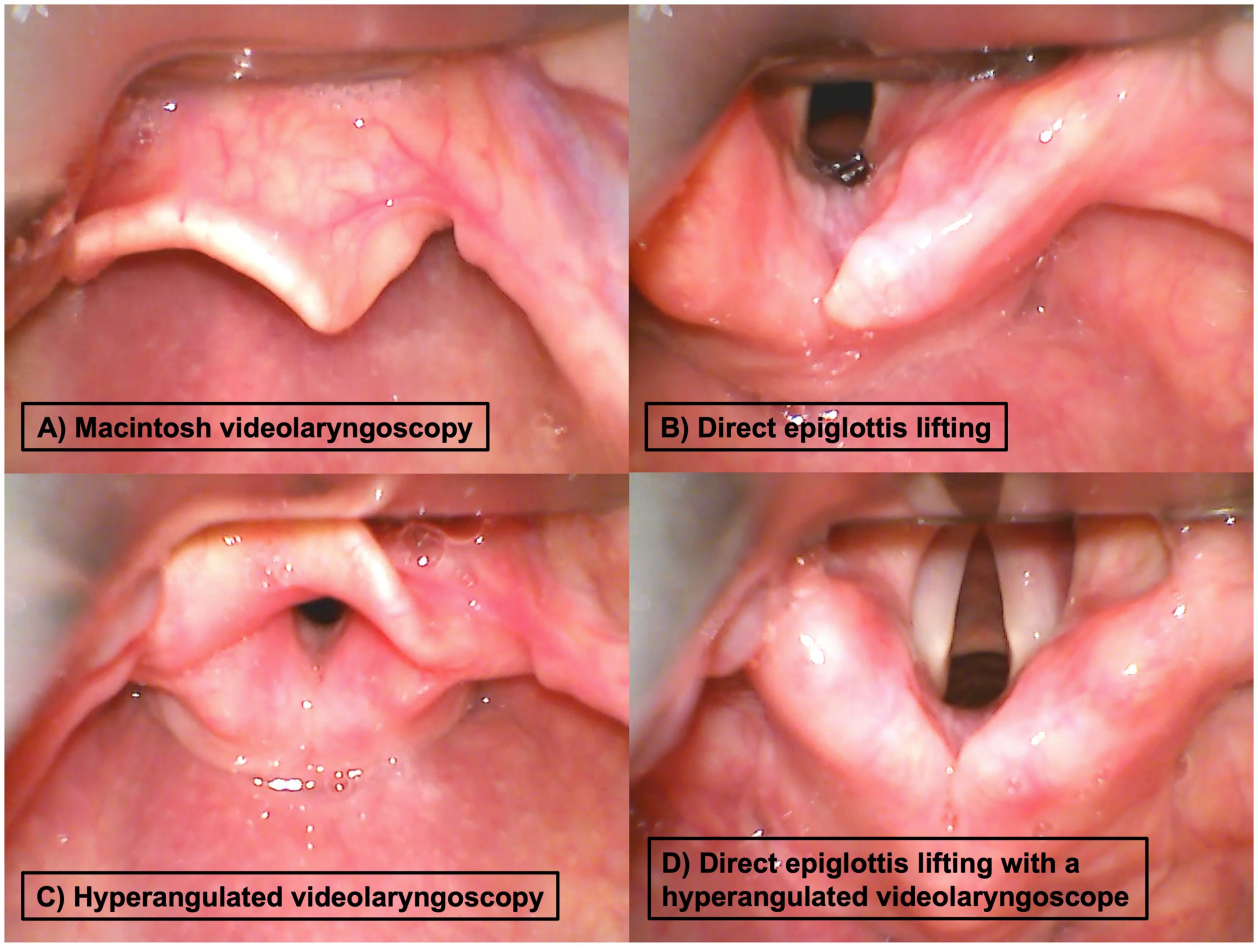
Examples for different glottis exposures during videolaryngoscopy (from the videolaryngoscopy camera perspective) during **(A)** Macintosh videolaryngoscopy (first-line technique in all patients) with the blade tip placed in the vallecula and possible optimization maneuvers, **(B)** direct epiglottis lifting, **(C)** hyperangulated blade with tip position in the vallecula, and **(D)** hyperangulated blade with direct epiglottis lifting.

Only patients with anticipated difficult airway management were included. Patients in whom awake tracheal intubation was planned or pregnant women were excluded. During the study period, study assessments and outcome variables were recorded separately from clinical notes to allow multiple independent assessments for participants who had multiple anesthetics. We only included cases where Macintosh videolaryngoscopy turned out to be difficult and the anesthetist decided on escalating by using a view optimization maneuver, either ‘direct epiglottis lifting with the Macintosh videolaryngoscope’, ‘conversion to hyperangulated videolaryngoscopy’, and/or ‘direct epiglottis lifting with a hyperangulated videolaryngoscope’ (Figure 1). The sequence of these escalation steps was noted and termed as second-line, third-line or fourth-line techniques.

### 2.3 Primary and secondary outcome measures

The primary outcome of this analysis is the improvement of the POGO (12) achieved by the applied view optimization maneuvers: (i) conversion from indirect to direct epiglottis lifting with the Macintosh videolaryngoscope; (ii) conversion from Macintosh to hyperangulated videolaryngoscopy; and (iii) conversion from Macintosh videolaryngoscopy to direct epiglottis lifting with a hyperangulated videolaryngoscope. The improvement of the glottis view grades achieved by these optimization maneuvers [six grades as previously reported (11), modified after (32–34); Table 1] is a secondary outcome measure.

**Table 1:** Grading of the glottis view gathered by videolaryngoscopy.

Glottis view grade*	Description
Grade 1 view	Vocal cords completely visible
Grade 2a view	Part of the cords visible
Grade 2b view	Posterior cords only just visible
Grade 2c view	Arytenoids but not cords visible
Grade 3 view	Epiglottis but no glottis visible
Grade 4 view	Laryngeal structures not visible

^*^Best view obtained with the videolaryngoscope camera as proposed by Petzoldt and coworkers et al. (10, 11) [modified after Cormack et al. (33), Yentis et al. (34), Cook et al. (32)].

### 2.4 Video analysis

During the study assessment, we tried to capture all videos gathered by videolaryngoscopy. Only integer videos that include the full sequences of all applied laryngoscopy maneuvers in a patient were considered for post-hoc video analysis, while invalid, interrupted or incomplete videos were excluded. Videos were analyzed and quantitative measures were performed using Datinf^®^ Measure 3 (Datinf^®^ GmbH, Tübingen, Germany). All videolaryngoscopy videos were split into the predefined sequences (Macintosh videolaryngoscopy, direct epiglottis lifting, hyperangulated videolaryngoscopy, direct lifting with a hyperangulated videolaryngoscope) and rated by three assessors (VAW, VK, MB) independently from each other. Assessors were instructed to rate the best view obtained within the corresponding video sequence, with or without backward upward rightward pressure, using POGO (12) and six glottis view grades (11) (Table 1). Unclear or borderline findings were additionally reviewed independently by two consultant anesthetists (PB, MP). All raters were blinded to the preoperative airway assessments, outcome measures, and the ratings of each other. Discrepancies were discussed thereafter, and a consensus vote was reached in each case.

### 2.5 Statistical methods

The primary hypothesis states that the improvement of the POGO (12) achieved by direct epiglottis lifting is non-inferior to the one accomplished by a conversion to hyperangulated videolaryngoscopy in cases where Macintosh videolaryngoscopy turned out to be difficult. A non-inferiority margin of 10% was considered clinically relevant (13, 19). The improvement of the glottis view grade (six grades, Table 1) achieved by these view optimization maneuvers was evaluated in a secondary analysis.

For descriptive statistics, sample characteristics are given as absolute and relative frequencies, mean (%) as well as median (IQR) whichever is appropriate.

To test the primary hypothesis, we used a linear mixed regression model. For the secondary analysis, we applied a linear regression model. The dependent variable was the continuous difference of the POGO or glottis view grade between the initial technique and subsequent optimization maneuver. Optimization maneuvers were included as independent variable with the categories: (A) conversion from indirect to direct epiglottis lifting with the Macintosh videolaryngoscope, (B) conversion from Macintosh to hyperangulated videolaryngoscopy, and (C) conversion from Macintosh videolaryngoscopy to direct epiglottis lifting with a hyperangulated videolaryngoscope with (A) being the reference category.

The consensus values of the POGO or glottis view grades with Macintosh videolaryngoscopy were used for baseline adjustment. In the mixed model, a random intercept was modeled for each patient to account for repeated measurements. For the secondary analysis, a linear regression model was modelled as the random effect variance was estimated to zero. The improvements of the POGO and glottis view grade achieved by the corresponding view optimization maneuvers were estimated with marginal means with 95% confidence intervals. Contrasts between the estimated marginal means with 95% confidence intervals were used for further pairwise comparisons between optimization maneuvers. All confidence intervals were calculated using the method of Satterthwaite to calculate degrees-of-freedom and standard errors.

The two-sided significance level is set to 5%. The primary hypothesis is significant if the lower bound of the 95% confidence interval of the contrast in POGO improvement between direct epiglottis lifting and hyperangulated videolaryngoscopy is larger than the negative non-inferiority margin of −10%. Statistical analysis was performed using SPSS statistics version 25 (IBM Inc., Armonk, NY, USA) and R version 4.1.3 (R Foundation for Statistical Computing, Vienna, Austria).

## 3 Results

**Figure 2:**
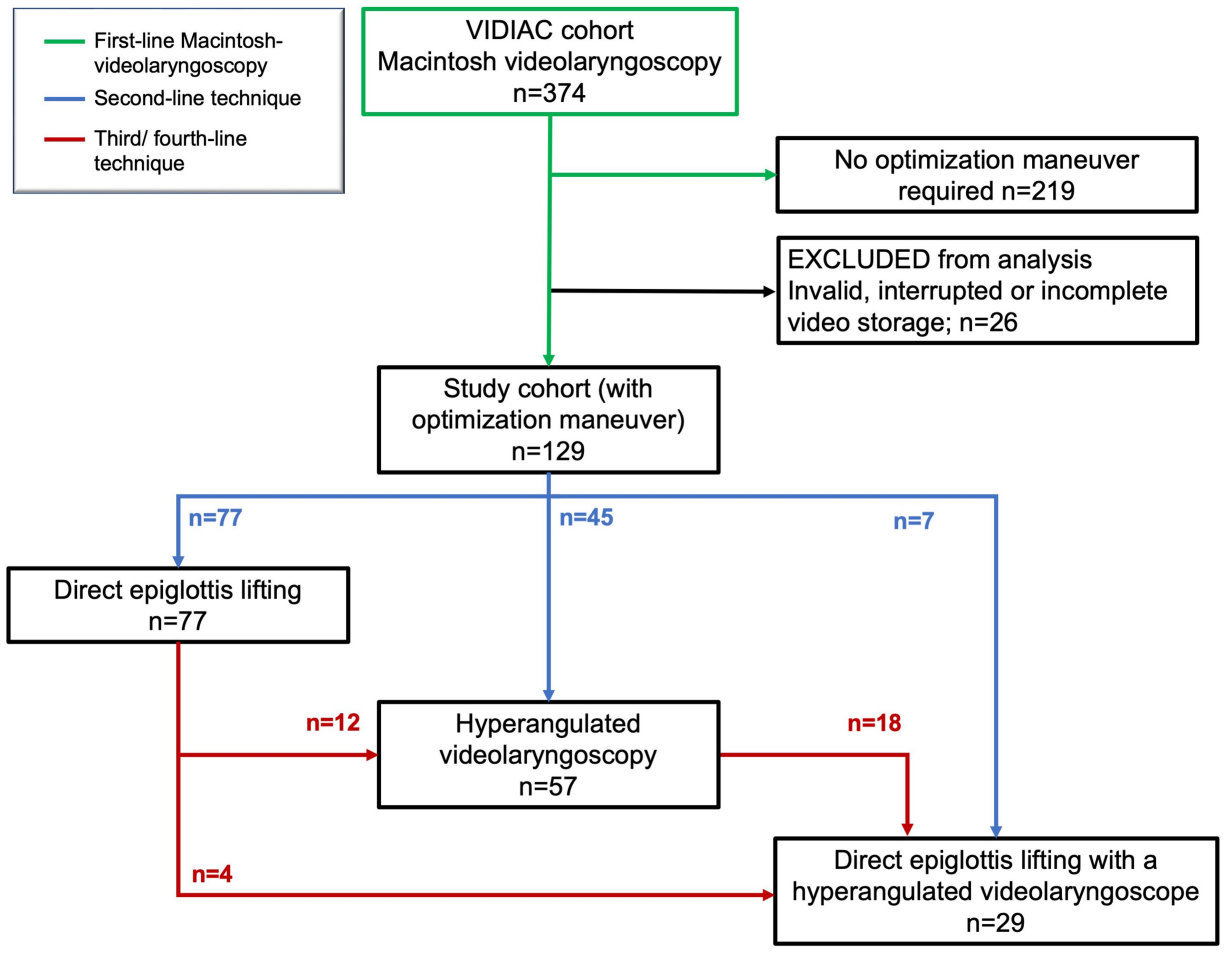
Study flow and sequential use of view optimization techniques; first-line Macintosh videolaryngoscopy (green), second-line technique (blue), third/fourth-line technique (red).

In the VIDIAC study, 374 anesthetics in 320 participants with expected difficult airway management who were managed using Macintosh videolaryngoscopy were included. The present non-inferiority analysis comprises 129 anesthetics in 107 participants with difficult Macintosh videolaryngoscopy, in whom 163 second, third, and fourth-line optimization techniques (77 direct epiglottis lifting, 57 hyperangulated videolaryngoscopy, 29 direct epiglottis lifting with hyperangulated videolaryngoscopes) were employed in various sequential orders (Figure 2). Meanwhile, 26 anesthetics could not be considered for analysis due to invalid, interrupted or incomplete video storage. Baseline characteristics are given in Table 2.

**Table 2:** Baseline characteristics.

Variables		Successful Macintosh videolaryngoscopy (219 cases)*	Difficult Macintosh videolaryngoscopy (129 cases)*
Age (years), mean ± SD		60.36 ± 14.57	63.16 ± 12.02
Body mass index (kg m^−2^), mean ± SD		26.73 ± 6.68	25.20 ± 5.92
Sex, *n* (%)	Male	148 (67.6)	94 (72.9)
	Female	71 (32.4)	35 (27.1)
ASA physical status, class, *n* (%)	I	15 (6.9)	4 (3.1)
	II	88 (40.2)	35 (27.1)
	III	109 (49.8)	86 (66.7)
	IV	7 (3.2)	4 (3.1)
Mallampati score, *n* (%)	I	32 (14.6)	9 (7.0)
	II	53 (24.2)	24 (18.6)
	III	80 (36.5)	33 (25.6)
	IV	54 (24.7)	63 (48.8)
Previous, *n* (%)	Neck dissection	47 (21.5)	55 (42.6)
	Tracheostomy	37 (16.9)	56 (43.4)
	Neck radiotherapy	34 (15.5)	47 (36.4)
	Mouth floor resection	20 (9.1)	29 (22.5)
Supraglottic tumors, *n* (%)		62 (28.3)	25 (19.4)
Glottis tumors, *n* (%)		25 (11.4)	10 (7.8)
Simplified airway risk index, mean ± SD		3.84 ± 2.22	5.56 ± 2.78
Could not bite upper lip, *n* (%)		66 (30.1)	60 (46.5)
Type of surgery, *n* (%)	Laryngopharyngeal	100 (45.7)	41 (31.8)
	Lower Jaw	47 (21.5)	36 (27.9)
	Neck, maxillofacial	44 (20.1)	26 (20.2)
	Ear, Nose	19 (8.7)	13 (10.1)
	Dentoalveolar	9 (4.1)	13 (10.1)
Nasal intubation, *n* (%)		56 (25.6)	51 (39.5)
Rapid sequence induction, *n* (%)		19 (8.7)	7 (5.4)
Difficult intubation^#^, *n* (%)		48 (21.9)	57 (44.2)
Difficult laryngoscopy^#^, *n* (%)		3 (1.4)	61 (47.3)
Time to tracheal intubation (min), mean ± SD		79.17 ± 91.57	221.77 ± 195.24
Airway related adverse events, *n* (%)		24 (11.0)	35 (27.1)

Values are number (proportion) or mean ± SD; *26 cases had to be excluded from the analysis due to invalid, interrupted or incomplete video storage; ASA, American Society of Anesthesiologists; ^#^ as defined by the 2022 ASA guidelines (5).

In the studied cohort of patients, vocal cords were not visible (grade 2c view or worse (11), Table 1) during the initial Macintosh videolaryngoscopy in 118/129 (91.5%) of the cases and in 19/77 (24.7%) after direct epiglottis lifting, in 21/57 (36.8%) after conversion to hyperangulated videolaryngoscopy and in 0/29 (0%) after direct epiglottis lifting with a hyperangulated videolaryngoscope (Table 3). The mean ± SD POGO (%) was 4.5 ± 17.5 for Macintosh videolaryngoscopy, 54.3 ± 37.3 for direct epiglottis lifting, 46.9 ± 41.4 for hyperangulated videolaryngoscopy and 74.7 ± 24.0 for direct epiglottis lifting with a hyperangulated videolaryngoscope.

**Table 3:** Glottis view grades and POGO with Macintosh videolaryngoscopy and different optimization maneuvers.

	Macintosh videolaryngoscopy (*n* = 129)	Direct epiglottis lifting (*n* = 77)	Hyperangulated videolaryngoscopy (*n* = 57)	Direct epiglottis lifting with a hyperangulated videolaryngoscope (*n* = 29)
Grade 1 glottis view (%)	2 (1.6)	17 (22.1)	9 (15.8)	9 (31.0)
Grade 2a glottis view (%)	1 (0.8)	24 (31.2)	22 (38.6)	12 (41.4)
Grade 2b glottis view (%)	8 (6.2)	17 (22.1)	5 (8.8)	8 (27.6)
Grade 2c glottis view (%)	24 (18.6)	18 (23.4)	5 (8.8)	0
Grade 3 glottis view (%)	77 (59.7)	1 (1.3)	14 (24.6)	0
Grade 4 glottis view (%)	17 (13.2)	0	2 (3.5)	0
Vocal cords not visible (grade 2c view or worse) (%)	118 (91.5)	19 (24.7)	21 (36.8)	0
POGO ^#^ (%), mean ± SD	4.53 ± 17.5	54.33 ± 37.25	46.86 ± 41.4	74.66 ± 23.99
Improvement of POGO compared to Macintosh videolaryngoscopy (%), mean ± SD	-	50.64 ± 39.10	41.59 ± 41.01	71.56 ± 26.28
Improvement of glottis view compared to Macintosh videolaryngo-scopy (grades), mean ± SD	-	2.1 ± 1.2	1.9 ± 1.4	3.2 ± 0.9

POGO, percentage of glottic opening.

### 3.1 Improvement of POGO and glottis view grade

**Figure 3:**
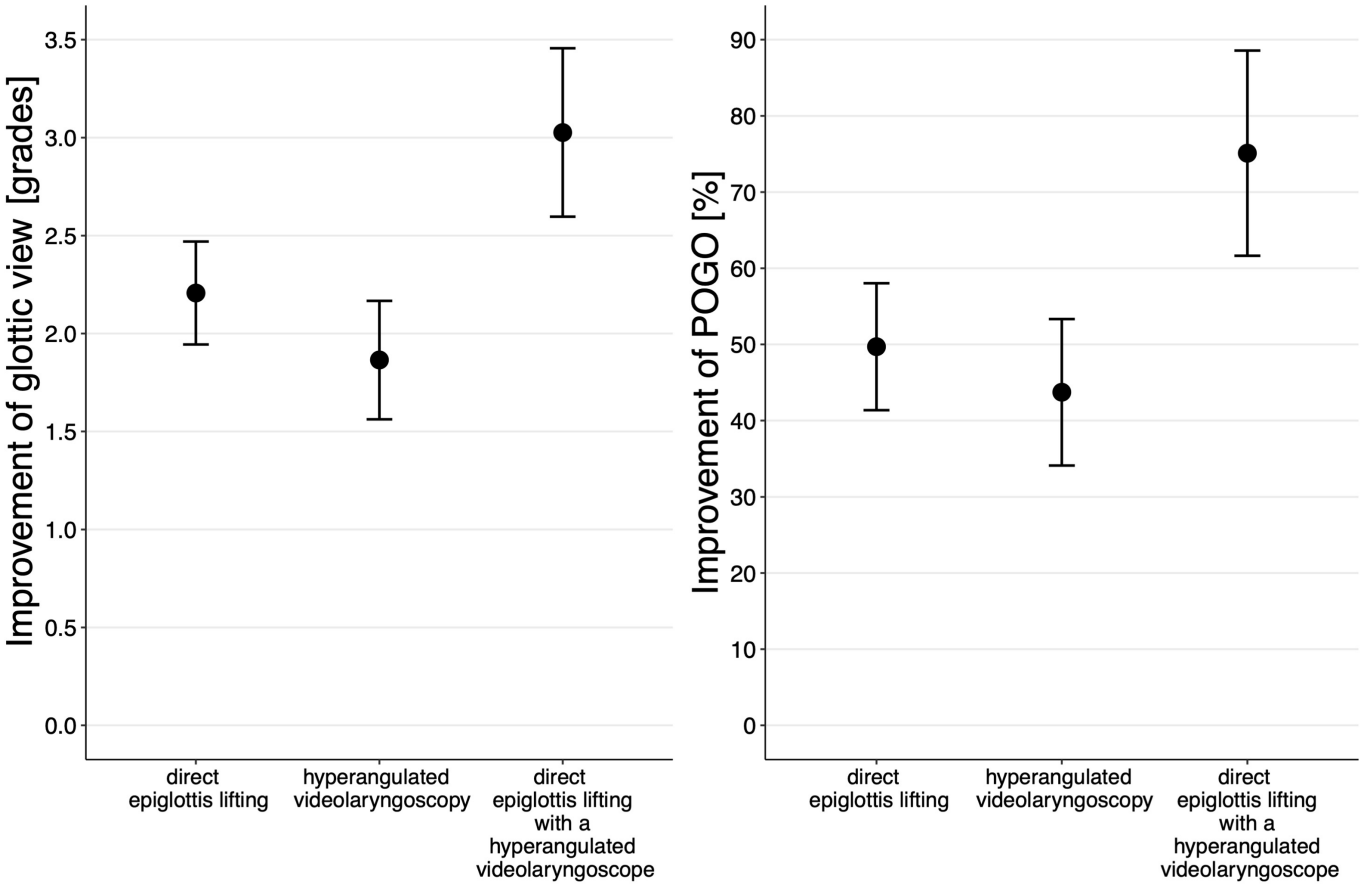
Estimated marginal means (95% CI) for improvement in POGO and glottis view grades (Table 1) for applied view optimization maneuvers.

Supplementary Table S1 gives the results of the regression models estimating the effects of different view optimization maneuvers. The estimated marginal means show that direct epiglottis lifting improved POGO (mean; 95% CI) by +49.7% (41.4 to 58.0; *p* < 0.001) and the glottis view by +2.2 grades (1.9 to 2.5; *p* < 0.001), hyperangulated videolaryngoscopy improved POGO by +43.7% (34.1 to 53.3; *p* < 0.001) and glottis view by +1.9 grades (1.6 to 2.2; *p* < 0.001), and direct epiglottis lifting with a hyperangulated videolaryngoscope improved POGO by +75.1% (61.7 to 88.6; *p* < 0.001) and glottis view by +3.0 grades (2.6 to 3.5; *p* < 0.001) (Figure 3; Supplementary Table S2).

The contrasts of the marginal means that result from a pairwise comparison between different optimization maneuvers show a mean difference in POGO improvement between the ‘conversion from indirect to direct epiglottis lifting with the Macintosh videolaryngoscope’ and the ‘conversion from Macintosh to hyperangulated videolaryngoscopy’ of 6.0% (95% CI: −6.5 to 18.5%) (Supplementary Table S3). Referring to the primary hypothesis, we found the lower bound of the 95% CI value (−6.5%) to be larger than the negative non-inferiority margin of −10%. Hence, non-inferiority of the POGO improvement by the view optimization maneuver ‘conversion from indirect to direct epiglottis lifting with the Macintosh videolaryngoscope’ compared to ‘conversion to hyperangulated videolaryngoscopy’ was significantly confirmed.

## 4 Discussion

Our study confirmed that the view achieved by direct epiglottis lifting (+50% POGO improvement) was non-inferior to the one achieved by conversion to hyperangulated videolaryngoscopy (+44% POGO improvement) in situations where Macintosh videolaryngoscopy proved to be difficult. We further noticed a synergistic effect: Direct epiglottis lifting with hyperangulated blades yields a best glottis exposure (+75% POGO improvement).

Being non-inferior to the clinical standard, what argues for direct epiglottis lifting? Nowadays, Macintosh videolaryngoscopy is often liberally available at the bed-side in many departments and is often easily accessible even for less experienced airway operators. Dependent on the local protocols, hyperangulated blades are often preserved for the more experienced airway operators. Obviously, in situations in which Macintosh videolaryngoscopy is difficult, the blade is already *in-situ* by the time the airway operator starts to recognize that the view is relevantly restricted; hence transition to a different blade or devices is not required if direct epiglottis lifting is attempted for optimization. This, in turn, might save time and preserve health-care resources. View improvement with hyperangulated videolaryngoscopy has been reported to not necessarily translate into easier or faster intubation (15, 35), angle dissonance may occure and the tracheal tube might impinge at the anterior commissure or tracheal wall (36, 37). However, it is unknown if tube placement might also be altered due to direct epiglottis lifting with a Macintosh videolaryngoscope. Direct epiglottis lifting with a Macintosh blade is a quite simple technique, inspired by the straight blade technique (18, 20–23) facilitated by just a tiny additional movement of the blade tip within the ongoing laryngoscopy procedure. However, to the best of our knowledge, direct epiglottis lifting with Macintosh-type blades has not yet been recommended in guidelines and only very limited data exist (17, 19). However, in our opinion direct epiglottis lifting is only reasonable if applied to optimize a severely restricted glottis view (vocal cords not visible); here, the incompletely lifted epiglottis often acts like a shield in the camera view axis and therefore restricts the camera view on the glottis (11). In this situation, direct lifting might be particularly beneficial to improve glottis exposure (17, 19). Notably, it has to be considered that view improvement might not be expedient and desirable in some clinical situations as a deliberately restricted glottis view might translate into faster and easier tracheal intubation with a videolaryngoscope (35). Only a single study by Oh et al. (19) compared the glottis view gathered by indirect and direct lifting of the epiglottis with a Macintosh videolaryngoscope in 60 patients without expected airway difficulty and found a significant improvement of the glottis exposure by direct epiglottis lifting. However, in this study 98% of the patients had grade 1 or 2 glottis views (19).

But what argues for conversion to hyperangulated videolaryngoscopy to optimize the glottis exposure in situations where the glottis view with Macintosh videolaryngoscopy proves to be deficient? First of all, we did not observe a single case in which the glottis view declined after conversion to a hyperangulated blade in our analysis. Further, with hyperangulated blades the epiglottis can also be lifted directly and our data suggest that this escalation step most substantially exposes the glottis. It might be a reasonable strategy to skip intermediate escalation steps and to immediately convert to direct epiglottis lifting with a hyperangulated videolaryngoscope in some time-critical situations. Further direct epiglottis lifting with a hyperangulated videolaryngoscope appears to be the most reasonable first escalation step if hyperangulated videolaryngoscopy fails.

Notably, it is unknown if direct epiglottis lifting might mechanically alter the epiglottis; however, despite comprehensive experience with direct epiglottis lifting with the straight blade technique (18, 20–23), to the best of our knowledge, the issue of mechanical alterations of the epiglottis due to direct epiglottis lifting has not been reported in larger case series (18, 38).

Based on the existing evidence, none of the described methods can be particularly highlighted for daily clinical practice. The decision to use a particular view optimization maneuver in situations where Macintosh videolaryngoscopy proves to be difficult is context-dependent and relies on the available resources, equipment, time, preconditions, risk of hypoxia as well as the personal preference and skill level of the airway operator and airway team.

This study has some limitations: as it is a single-center trial and airway management, equipment, strategies as well as escalation and backup plans differ between departments and regions, findings should not be generalized or extrapolated to other institutions without appropriate cautions. Data were assessed in patients with anticipated difficult airways undergoing ear, nose, and throat or oral and maxillofacial surgery. The study was not randomized; however, it can be considered a strength of the study that data were assessed in real-world conditions. However, for the interpretation of our study findings, the preconditions, such as different skill levels of the airway operators and relevant number of nasal and rapid sequence intubations have to be considered.

In conclusion, our study demonstrates that conversion to hyperangulated videolaryngoscopy (+44% POGO improvement) as well as direct epiglottis lifting (+50% POGO improvement) effectively improved glottis visualization in situations where Macintosh videolaryngoscopy proved to be difficult. Direct epiglottis lifting was non-inferior to conversion to hyperangulated videolaryngoscopy, while the combination of both optimization maneuvers yielded the best glottis exposure (+75% improvement).

## Data availability statement

The raw data supporting the conclusions of this article will be made available by the authors, without undue reservation.

## Ethics statement

The studies involving humans were approved by Ethics Committee of the Medical Association of Hamburg (PV5856, August 10, 2018, amendment August 12, 2019, chair: Prof. Dr. R. Stahl). The studies were conducted in accordance with the local legislation and institutional requirements. The participants provided their written informed consent to participate in this study.

## Author contributions

VW: Conceptualization, Data curation, Formal analysis, Investigation, Methodology, Writing – original draft. VK: Conceptualization, Data curation, Formal analysis, Methodology, Writing – original draft. PB: Conceptualization, Project administration, Resources, Supervision, Writing – review & editing. MB: Data curation, Formal analysis, Investigation, Writing – original draft. PS: Conceptualization, Formal analysis, Writing – review & editing. HS: Conceptualization, Data curation, Writing – review & editing. AD: Supervision, Writing – review & editing. MS: Formal analysis, Methodology, Visualization, Writing – original draft. CZ: Conceptualization, Supervision, Writing – review & editing. MP: Conceptualization, Data curation, Formal analysis, Investigation, Methodology, Project administration, Supervision, Writing – review & editing.
