# Supplementary material for: Hyperangulated blades or direct epiglottis lifting to optimize glottis visualization in difficult Macintosh videolaryngoscopy: a non-inferiority analysis of a prospective observational study
Source: Front Med (Lausanne). 2023 Nov 30;10:1292056. doi: 10.3389/fmed.2023.1292056 (PMC10720620; doi:10.3389/fmed.2023.1292056)
Supplement: Supplementary file 1 [file Table_1.DOCX]

| **Supplemental Table 1:** Linear mixed regression (POGO) and linear regression (glottis view grades) to estimate the effects of different optimization maneuvers (reference method: direct epiglottis lifting) | | | | | | |
| --- | --- | --- | --- | --- | --- | --- |
|  | **Differences in POGO improvement**  (linear mixed regression^#^) observations *n*=163 | | | **Differences in glottis view grade improvement** (linear regression^##^) observations *n*=163 | | |
| Optimization maneuvers* | estimates | 95%-CI | *P*-value | estimates | 95%-CI | *P*-value |
| (Intercept) | 52.21 | 43.79 to 60.64 | <.001 | 0.06 | -0.94 to 1.07 | 0.9 |
| Conversion from Macintosh to hyperangulated videolaryngoscopy | -5.98 | -18.49 to 6.53 | 0.35 | -0.34 | -0.74 to 0.06 | 0.1 |
| Conversion from Macintosh videolaryngoscopy to direct epiglottis lifting with a hyperangulated videolaryngoscope | 25.40 | 9.79 to 41.01 | 0.002 | 0.82 | 0.31 to 1.33 | 0.002 |
| Glottis view grade with Macintosh videolaryngoscopy; consensus |  |  |  | 0.45 | 0.24 to 0.65 | <.001 |
| POGO with Macintosh videolaryngoscopy; consensus | -0.61 | -0.94 to -0.27 | <.001 |  |  |  |
| CI, confidence interval; analysis based on the data from 163 optimization maneuvers during 129 anesthetics in 107 patients; *the optimization maneuver conversion from indirect to direct epiglottis lifting with the Macintosh videolaryngoscope was used as reference category; consensus values of the POGO or the glottis view grades were used for baseline adjustment; ^#^ a random intercept was modeled for each patient to account for repeated measurements; random effects: residual within-patient variance (σ^2^): 1203.76, between patients variance (τ^00^): 127.03, ^##^ as the random effect variance was estimated to zero a linear regression model was modeled for glottis view grades; we did not adjust for gender, age and body mass index as we did not find a relevant influence | | | | | | |
