# Supplementary material for: Hyperangulated blades or direct epiglottis lifting to optimize glottis visualization in difficult Macintosh videolaryngoscopy: a non-inferiority analysis of a prospective observational study
Source: Front Med (Lausanne). 2023 Nov 30;10:1292056. doi: 10.3389/fmed.2023.1292056 (PMC10720620; doi:10.3389/fmed.2023.1292056)
Supplement: Supplementary file 2 [file Table_2.DOCX]

| **Supplemental Table 2:** Estimated marginal means | | |
| --- | --- | --- |
| **Optimization maneuvers** | **POGO improvement**  **(95% CI); *P*-value** | **Glottis view grade improvement (95% CI); *P*-value** |
| Conversion from indirect to direct epiglottis lifting with the Macintosh videolaryngoscope | 49.70 (41.36 to 58.04); *P* <.001 | 2.21 (1.94 to 2.47); *P* <.001 |
| Conversion from Macintosh to hyperangulated videolaryngoscopy | 43.72 (34.11 to 53.33); *P* <.001 | 1.87 (1.56 to 2.17); *P* <.001 |
| Conversion from Macintosh videolaryngoscopy to direct epiglottis lifting with a hyperangulated videolaryngoscope | 75.10 (61.65 to 88.56); *P* <.001 | 3.03 (2.60 to 3.46); *P* <.001 |
| POGO, percentage of glottic opening; CI, confidence interval | | |
