# Supplementary material for: Hyperangulated blades or direct epiglottis lifting to optimize glottis visualization in difficult Macintosh videolaryngoscopy: a non-inferiority analysis of a prospective observational study
Source: Front Med (Lausanne). 2023 Nov 30;10:1292056. doi: 10.3389/fmed.2023.1292056 (PMC10720620; doi:10.3389/fmed.2023.1292056)
Supplement: Supplementary file 3 [file Table_3.DOCX]

| **Supplemental Table 3:** Contrasts of marginal means; pairwise comparison between different optimization maneuvers | | |
| --- | --- | --- |
| **Contrasts between optimization maneuvers** | **Differences in POGO improvement (95% CI)** | **Differences in glottis view grade improvement (95% CI)** |
| Macintosh videolaryngoscopy to direct epiglottis lifting  versus  Macintosh videolaryngoscopy to hyperangulated videolaryngoscopy | 5.98 (-6.53 to 18.49) | 0.34 (-0.06 to 0.75) |
| Macintosh videolaryngoscopy to direct epiglottis lifting  versus  Macintosh videolaryngoscopy to direct epiglottis lifting with a hyperangulated videolaryngoscope | -25.40 (-41.02 to -9.79) | -0.82 (-1.33 to -0.31) |
| Macintosh videolaryngoscopy to hyperangulated videolaryngoscopy  versus  Macintosh videolaryngoscopy to direct epiglottis lifting with a hyperangulated videolaryngoscope | -31.38 (-47.48 to -15.29) | -1.16 (-1.69 to -0.64) |
| POGO, percentage of glottic opening; CI, confidence interval | | |
